# Draft-genome sequencing of multidrug-resistant hypervirulent *Klebsiella pneumoniae* from neonatal septicaemia at SRHU, Dehradun, India

**DOI:** 10.1128/mra.00997-25

**Published:** 2026-03-24

**Authors:** Indrani Sarkar, Arpana Singh, Mousumi Hazra, Debarati Chattopadhyay, Uttam Kumar Nath, Barnali Kakati, Saugata Hazra

**Affiliations:** 1 Department of Biosciences and Bioengineering, Indian Institute of Technology-Roorkee, Roorkee, Uttarakhand, India; 2 Department of Medical Oncology and Haematology, All India Institute of Medical Sciences, Rishikesh, Uttarakhand, India; 3 Department of Microbiology, Swami Rama Himalayan University 422223, Dehradun, Uttarakhand, India; 4 Department of Botany and Microbiology, Gurukula Kangri (Deemed to be University) 123264, Haridwar, Uttarakhand, India; 5 Department of Burn and Plastic Surgery, All India Institute of Medical Sciences, Rishikesh, Uttarakhand, India; 6 Department of Microbiology, All India Institute of Medical Sciences Rishikesh, Rishikesh, Dehhradun, India; 7 Centre for Nanotechnology, Indian Institute of Technology-Roorkee, Roorkee, Uttarakhand, India; Queens College, Queens, New York, USA

**Keywords:** *Klebsiella*, MDR, hypervirulent, India, *de novo *sequencing

## Abstract

Multidrug resistance makes the Gram-negative bacterium *Klebsiella pneumoniae* a growing threat and a major contributor to neonatal sepsis. At SRHU, Dehradun, India, we discovered four hypervirulent multidrug-resistant strains from bloodstream infections in neonates, highlighting their clinical significance and treatment and infection control implications.

## ANNOUNCEMENT

*Klebsiella pneumoniae*, a non-motile, rod-shaped, Gram-negative bacterium, is a major cause of nosocomial infections, particularly in immunocompromised patients (1). In this study, we present the *de novo* draft-genome sequencing of multidrug-resistant, hypervirulent *Klebsiella* strains isolated from the blood samples of neonates with sepsis in India. We collected blood samples from suspected sepsis patients in the neonatology ICU at SRHU, Dehradun. These samples were placed in pediatric BacT/Alert blood culture bottles and sent to the Department of Microbiology for analysis. All isolates included in the study were collected from clinical samples received in the Microbiology laboratory during the defined study period (2023–2024). The bottles were incubated at 37°C. Upon flagging positive for microbial growth, we immediately performed direct microscopy with Gram staining, which revealed characteristic Gram-negative, short, plump, capsulated bacilli. Subcultures were then prepared on 5% sheep blood agar and MacConkey agar (MA) and aerobically incubated at 37°C for 18–24 h. The observation of lactose-fermenting, mucoid colonies on MA strongly suggested the presence of a *Klebsiella* species. Further, 16s rRNA sequencing was performed for taxonomic confirmation. Antibiotic susceptibility testing was done using the Vitek 2 automated system in the Microbiology laboratory at Himalayan Institute of Medical Sciences, SRHU, Dehradun, and interpreted as per CLSI standard guidelines (2). For molecular analysis, DNA was isolated from a single colony using an Xploregen DNA isolation kit. We precisely quantified the DNA using a QUBIT 3 Fluorometer and dsDNA HS dye. Subsequently, we prepared the samples for sequencing using the Kappa DNA library preparation kit. This involved enzymatically fragmenting 100 ng of intact DNA into 233–300 bp pieces. These fragments underwent end repair to create blunt ends, followed by adenylation with a single “A” nucleotide at the 3' ends and ligation with loop adapters. After adapter cleavage by the USER enzyme, the materials were purified using AMPure beads. Final PCR amplification (six cycles) was performed with NEBNext Ultra II Q5 Master Mix, Illumina universal primers, and sample-specific octamer primers. The sequencing was performed using the Illumina NovaSeq 6000 platform with 151 bp paired-end. The generated sequence data underwent rigorous quality assessment using FastQC v0.12.1 (3), and Trim Galore was used for adapter trimming. Raw reads were assembled with Unicycler v0.5.0 (4), and the resulting sequences were further processed by Medusa 2.0 (5) to ensure high-quality outcomes. We determined genome completeness using BUSCO 5.8.2 (6). For genome annotation, we utilized NCBI-PGAP (7). Finally, ResFinder 4.7.2 (8) and PlasmidFinder 2.1 (9) were employed to identify antimicrobial resistance genes and plasmids within the secondary assembly data. K-typing was performed using Pathogenwatch (https://pathogen.watch/). All these tools were used with default parameters. Table 1 lists the overall genomic statistics of the sequenced genomes.

**TABLE 1:** An overview of the genome statistics for the four sequenced genomes

Genome name	No. of reads	Genome completeness	Genome coverage	N50	GC%	Predicted resistant antibiotics	Plasmid types
HIMS_HL_K004	6.4M	98.9	169.12×	5,372,713	54	Aztreonam, cefepime, cefixime, cefotaxime, cefoxitin, ceftazidime, ceftriaxone, cephalothin	IncFIB(pKPHS1) IncFIB(pNDM-Mar) IncFII IncHI1B(pNDM-MAR) IncR
HIMS_HL_K013	6.2 M	98.7	174.23×	5,322,099	57	Cefepime, cefixime, cefotaxime, cefoxitin, ceftazidime, cephalothin, amoxicillin, amoxicillin + clavulanic acid, ampicillin, ampicillin + clavulanic acid, meropenem, piperacillin, piperacillin + tazobactam	ColKP3 IncFIA IncFIB(pB171) IncFII
HIMS_HL_K019	7.6M	98.9	212.56×	5,288,603	57	Amoxicillin, amoxicillin + clavulanic acid, ampicillin, ampicillin + clavulanic acid, aztreonam, cefepime, cefixime, cefotaxime, cefoxitin, ceftazidime, ceftriaxone, cephalothin, imipenem, meropenem, piperacillin, piperacillin + tazobactam	ColKP3 IncFII
HIMS_HL_K020	5.4M	98.5	152.41×	5,275,872	58	Amoxicillin, amoxicillin + clavulanic acid, ampicillin, ampicillin + clavulanic acid, aztreonam, cefepime, cefixime, cefotaxime, cefoxitin, ceftazidime, ceftriaxone, cephalothin, ertapenem, meropenem, piperacillin, piperacillin + tazobactam	ColKP3 IncFII

Both HIMS_HL_K004 and HIMS_HL_K013 possess K-antigen type K-64 with bla-TEM, bla-NDM, and bla-CTX-M. HIMS_HL_K019 and HIMS_HL_K020 were found to be K2 type with bla-TEM1B, bla-NDM5, and bla-SHV106. K2 is globally prevalent and highly virulent, and type K64 is regionally prevalent and/or associated with emerging threats due to its linkage with carbapenem resistance.

## Data Availability

These genomes are available under BioProject PRJNA1264554. GenBank accession numbers and BioSample numbers are HIMS_HL_K004 (JBOCYP000000000; SAMN48564314), HIMS_HL_K013 (JBOCYG000000000; SAMN48564323), HIMS_HL_K019 (JBOCYC000000000; SAMN48564327), and HIMS_HL_K020 (JBOCYB000000000; SAMN48564328).
